# Tool, Threat, Tutor, Talk, and Trend: College Students’ Attitudes toward ChatGPT

**DOI:** 10.3390/bs14090755

**Published:** 2024-08-27

**Authors:** Sen-Chi Yu, Yueh-Min Huang, Ting-Ting Wu

**Affiliations:** 1 Department of Counseling and Applied Psychology, National Taichung University of Education, Taichung 403, Taiwan; 2 Department of Engineering Science, National Cheng Kung University, Tainan 701, Taiwan; 3 Graduate School of Technological and Vocational Education, National Yunlin University of Science and Technology, Yunlin 640, Taiwan; ttwu@yuntech.edu.tw

**Keywords:** ChatGPT, artificial intelligence, attitude, chatbot, gender differences

## Abstract

The purposes of this study are to investigate college students’ attitudes toward ChatGPT and to understand whether gender makes any difference in their attitudes. We developed the ChatGPT attitude scale (CAS) and administrated it to a sample of 516 Taiwan college students. Through an exploratory factor analysis, the 5-T (Tool, Tutor, Talk, Trend, and Threat) model of CAS was extracted and validated via confirmatory factor analysis. The CAS exhibited good reliability and validity and can be used to explain ChatGPT attitudes. According to our findings, university students consider ChatGPT an important “Tool” in their daily life. Additionally, ChatGPT plays a significant “Tutor” role, assisting with language translation and knowledge learning. Besides its utilitarian functions, ChatGPT also serves as a “Talk” feature, offering interactive chat and emotional support. Currently, students also acknowledge ChatGPT as an important “Trend” of the times, but they are also deeply concerned about the potential “Threat” of content falsification and job displacement brought on by ChatGPT. In terms of gender differences, compared to females, males scored higher than females in the total scale and in the Tool, Tutor, and Trend subscales. However, there was no significant difference between males and females in the Talk and Threat subscales. This gender difference result differs from previous research on robots or social media.

## 1. Introduction

In recent years, the use of chatbots or generative artificial intelligence (AI) applications, such as ChatGPT (Chat Generative Pre-Trained Transformer), has become increasingly popular [1]. ChatGPT, developed by OpenAI, is an AI chatbot program designed to simulate human conversation. It can generate human-like text and is capable of handling complex language tasks, including automatic text generation, question-answering, automatic summarization, and image generation. Since its launch in November 2022, ChatGPT has gained rapid popularity among users. According to a survey conducted in July 2023, ChatGPT surpassed one million users in its first week and accumulated over a billion users within two months, making it the fastest-growing consumer application in history. The ChatGPT historical page has been visited over 9 billion times. In total, 12.31% of ChatGPT users are from the United States. Among ChatGPT users, 59.67% are male and 40.33% are female. Additionally, 53% of readers are unable to distinguish whether speed-reading articles were written by ChatGPT or a human [2].

The use of ChatGPT has had a significant impact on the academic and daily lives of university students [1,3]. ChatGPT can be utilized to create personalized educational materials and customized learning plans tailored to the unique needs and interests of each student [4]. Moreover, ChatGPT is an important assistant for university students in organizing information, learning foreign languages, and translation [3,5]. In addition to its academic applications, ChatGPT also influences students’ daily lives. Many people use ChatGPT to plan their travel itineraries, search for food, or obtain related lifestyle information. Although the use of ChatGPT is becoming increasingly popular among university students, there is still limited research on the psychological aspects and attitudes towards the use of ChatGPT.

Attitude is defined as a psychological tendency that is expressed by evaluating a particular entity with some degree of favor or disfavor [6]. People’s overall attitudes towards ChatGPT are likely to play a significant role in their acceptance of this AI technology. Previous research has found that students with more positive attitudes toward technology (e.g., computers and the Internet) are more likely to perceive technology-based learning tasks as interesting and important [7]. Therefore, it can be inferred that attitudes towards ChatGPT may have an impact on learning, although there is still limited research on this topic [5]. In the past, many studies have investigated ‘Internet attitudes” (e.g., [7,8], “robot attitudes” (e.g., [9]) or “AI-attitude” (e.g., [10]). However, the attitudes toward Chabot/ChatGPT are still limited, and most of the relevant studies are based on the technology acceptance model (TAM) [11,12,13,14]. College students are an important user group of ChatGPT. Understanding their attitudes toward ChatGPT is crucial for understanding the acceptance, usage behavior, and potential psychological consequences of this technology. Additionally, previous research has found gender differences in attitudes towards technology (e.g., [14,15]). Whether the same applies to ChatGPT attitudes remains to be studied. To summarize, there are three purposes behind this study. The first is to investigate the usage behavior and satisfaction of university students with ChatGPT. The second purpose of this study is to develop and validate the ChatGPT attitude scale. The third purpose of this study is to examine whether gender makes any difference in ChatGPT attitudes. This study’s specific research questions are as follows:

RQ1. What are the usage time/frequency and satisfaction of university students with ChatGPT?

RQ2. Does ChatGPT attitude scale developed by this study exhibit validity and reliability?

RQ3. Does gender of college students make any difference in their attitudes toward ChatGPT?

## 2. Literature Review

Research on ChatGPT attitude is still rare. Previous studies have shown that attitudes towards different information technology use (e.g., computers and the Internet) are related to each other [7,8,16]. Therefore, this study starts with the relevant research on computer/Internet-related attitudes and integrates research on chatbot and robot AI-related attitudes and finally summarizes the possible factors behind ChatGPT attitude.

### 2.1. Studies on Computer/Internet-Related Attitudes: 4T, 5T, and 6T Models

Taylor proposed the 3T model to measure computer/Internet-related attitudes, which are Tutor, Tool, and Tutee. “Tutor” refers to the computer acting as an expert in various fields to teach users. “Tool” refers to the computer being a tool that can greatly enhance efficiency. “Tutee” refers to the computer role-playing as a student [17].

To measure adolescents’ attitudes towards the Internet, Tsai modified Taylor’s 3T model of computer attitudes and proposed a 4T framework: Technology, Tool, Toy, and Travel [8]. “Technology” represents how technology strongly changes people’s lifestyles. “Tool” represents how the medium enables humans to accomplish various tasks to satisfy their needs. “Toy” represents joy, entertainment, and the continuously evolving and appealing nature of activities such as online gaming. “Travel” represents the Internet as a navigation tool that takes us to different places and websites, similar to traveling to different destinations.

Chou and his colleagues conducted a series of studies on students’ attitudes toward the Internet [16,18,19]. Chou et al. proposed a 4T framework, named “Tool, Toy, Telephone, and Treasure of Information”, to categorize people’s attitudes towards the Internet [18]. Subsequently, due to the rapid development of blogs and fan pages, Chou et al. added the “Territory” factor, creating a 5T framework [7]. Later, Chou and his colleagues pointed out that a substantial proportion of people engage in commercial activities and online shopping through the Internet [19]. Therefore, they added the “Trade” factor to the original 5T framework, resulting in a 6T model.

### 2.2. Studies on Chatbot/AI-Related Attitudes

Schepman and Rodway developed the General Attitudes towards Artificial Intelligence Scale (GAAIS) to measure AI attitudes [10]. Their factor analysis results revealed that GAAIS consists of two factors: positive and negative. The positive factor includes beliefs that AI is a beneficial tool, represents future trends, and provides new economic opportunities, among others. On the other hand, the negative factor reflects attitudes of worry, lack of control, perceived evil, and mistrust towards AI. Participants showed positive attitudes towards AI applications involving big data, such as astronomy, law, and pharmacology. However, they held negative attitudes towards applications involving human judgement tasks, such as healthcare and psychological counseling.

Koverola et al. developed the General Attitudes Towards Robots Scale (GAToRS), which consists of four dimensions based on individual/social levels and positive/negative influences [9]. The positive dimension includes feeling comfortable and pleasant around robots; having reasonable hopes for the overall development of robots; perceiving robots as important tools that assist humans; and the belief that if robots have emotions, one can be friends with them. The negative influence dimension includes feeling uneasy and anxious around robots and concerns about the overall development of robots.

Sindermann et al. developed the Attitude Towards Artificial Intelligence (ATAI) scale, which consists of five items. The participants were from Germany (N = 461; 345 female), China (N = 413; 145 female), and the UK (N = 84; 65 female) [20]. A factor analysis revealed that the ATAI scale includes two factors: acceptance and fear. The acceptance factor comprises items related to trusting artificial intelligence and perceiving it as a beneficial tool for humans. The fear factor includes items related to feeling afraid of AI and having concerns about AI potentially destroying humanity and causing mass unemployment. Regarding gender differences, males showed greater interest in technology and held more positive attitudes towards technology.

Li’s General attitudes toward ChatGPT scale [21], a five-item measure adapted from the the Attitude Toward Artificial Intelligence (ATAI) scale [20]. included two subscales–acceptance and fear of ChatGPT. Sample statements include “I trust ChatGPT.” (Acceptance) and “ChatGPT will cause many job losses” (fear).

Iqbal et al. conducted a qualitative research study to understand teachers’ perspectives on ChatGPT [22]. Semi-structured interviews were conducted with 20 university professors from a university in Pakistan. That study found that some professors had negative views and attitudes towards the use of ChatGPT. They expressed concerns such as the potential for students to use ChatGPT for cheating or plagiarism, as well as the possibility of it causing disruptions in the classroom. However, there were also those who held positive attitudes towards ChatGPT. They believed that ChatGPT could be a useful tool in certain situations, such as providing automatic replies to students and allowing teachers to focus more on other aspects of teaching. Additionally, ChatGPT was seen as having the potential to improve student engagement and motivation.

### 2.3. Frameworks for Studying ChatGPT Attitudes

The measurement tool for ChatGPT attitudes is still under development. Therefore, this study is based on the 5T/6T model of Internet attitudes, integrating research related to chatbots and AI attitudes, and proposes the following hypothetical 5T framework for studying ChatGPT attitudes:

(1) Tool

The dimension referred to as “Tool” in this study focuses on the functionality provided by ChatGPT, such as language translation, data collection, and organization. This dimension can be traced back to computer/Internet-related attitudes [8,17,18]. Studies on Internet attitudes also recognize the role of the Internet in information search and other functionalities, which are also present in AI/ChatGPT today, including personal assistants, travel planning, language translation, etc. [23]. Brandtzaeg and Følstad also mentioned that the purpose of using chatbots is to obtain necessary information and assistance for efficiently completing tasks [24]. The dimension of “Tool” is also found in other scales such as Koverola et al.’s “General Attitudes Towards Robots Scale” [9], Sindermann et al.’s “Attitude Towards Artificial Intelligence scale” [20], Schepman and Rodway’s “General Attitudes towards Artificial Intelligence Scale” [10], and Li’s “General attitudes toward ChatGPT scale” [21].

(2) Tutor

The dimension referred to as “Tutor” in this study highlights the learning guidance and feedback provided by AI. AI is widely used in tasks such as homework assistance, language translation, language learning, and presentation creation, acting as a digital teacher [1,3,25]. Iqbal et al. also identified the potential benefits of using ChatGPT to assist teaching, such as enhancing student engagement and motivation [22]. AI has numerous applications and explorations in the field of education [26]. ChatGPT can provide personalized learning materials and resource recommendations, and learners can utilize ChatGPT to reflect on their progress and learning, supporting autonomous learning [4]. Deveci Topal et al. applied chatbots in a science curriculum and found that students perceived them as useful, interesting, and providing helpful assistance for learning outside the classroom [27].

(3) Threat

In this study, the “Threat” dimension refers to the cognitive assessment and emotional response to the potential threat of ChatGPT. The cognitive aspect involved potential risks associated with ChatGPT, including concerns about job displacement, data privacy breaches, deception, and changes in interpersonal interactions. People worried that ChatGPT might be used as a weapon of mass deception, which could aid in deception-related crimes [28]. As AI technologies, including ChatGPT, continue to advance in their understanding, learning, and problem-solving capabilities, humans perceive potential threats. People also worry about job opportunities being replaced by AI [29,30]. Additionally, concerns exist regarding the impact of AI on our lifestyle and societal norms [31]. Research by Fast and Horvitz found that worries about the potential uncontrollability and ethical aspects of AI development are increasing [32]. Furthermore, AI raises concerns about related personal privacy issues. AI technologies require the collection and storage of data from the environment for learning and updating, potentially posing risks of privacy breaches [33]. The “Threat” factor also appears in other AI attitude-related measurement tools, such as Koverola et al., Sindermann et al., Schepman and Rodway, and Li [9,10,20,21].

The emotion response to the potential threat of ChatGPT involved negative emotions such as fear, anxiety, and distrust towards ChatGPT. People fear the rapid and uncontrollable development of AI technology. Anxiety about AI-related technologies is common in studies and scales related to AI and robots. For example, the GAAIS scale by Schepman and Rodway includes attitudes towards AI being worrisome, out of control, evil, and untrustworthy [10]. The ATAI scale by Sindermann et al. includes topics such as finding AI terrifying and the belief that AI will destroy humanity [20]. Koverola et al. included concerns about feeling uneasy and anxious about robots and overall worries about the development of robots [9]. In the educational context, Iqbal et al. found that university teachers expressed distrust in using ChatGPT, fearing that students might use it for cheating [22]. These studies reflect people’s fears and concerns about AI-related technologies.

(4) Talk

The dimension referred to as “Talk” in this study focuses on the ability to have conversations with chatbots and share psychological distress. The recent developments in GPT have made chatbots indistinguishable from humans [34]. This dimension is an important feature of chatbots. Some people find chatbots interesting and enjoyable, using them to pass the time [24]. Some social chatbot applications, such as Replika, have gained popularity for providing emotional and social support, offering companionship [35]. Deveci Topal et al. applied chatbots in science courses and found that students found chatting with chatbots to be fun [27]. For individuals who struggle with social interactions, chatbots can help reduce feelings of loneliness or fulfill their desired social patterns [24]. In the study by Bae Brandtzæg et al., nearly half of the participants reported receiving emotional support from the chatbot Woebot, highlighting the importance of the “Talk” function in ChatGPT [36].

(5) Trend

The dimension referred to as “Trend” in this study focuses on the trend and new era brought about by the development of ChatGPT. Despite the various challenges associated with ChatGPT, there is no doubt that artificial intelligence has become a driving force for innovation and revolution in various fields [37,38]. Artificial intelligence represents a trend of the new generation and the development achievements of emerging technologies. This factor also appears in the research by Schepman and Rodway and by Iqbal et al. [10,22].

Based on the six theoretical frameworks mentioned above, this study developed a preliminary version of the CAS scale consisting of 21 questions, which was then tested and evaluated for its validity and reliability

### 2.4. Gender Differences in Chatbot/AI-Related Attitudes

Research on AI attitudes has shown that, in terms of the overall score on the AI attitude scale, men tend to have more positive attitudes towards AI compared to women. However, the gender differences in specific dimensions of AI attitudes may not always be significant.

Grassini developed the AI Attitude Scale (AIAS) and found that female participants scored lower than male participants, indicating that men have more positive attitudes towards AI technology [15]. Similarly, the study by Pinto dos Santos et al. also found similar results [35]. They investigated the attitudes of medical students towards AI in the medical field and found that men had more positive attitudes and lower levels of fear towards AI. Another study by Lee and Yen explored gender differences in attitudes towards robots and found that men had a higher acceptance of robot services compared to women [39]. Men also preferred robot services over human services.

When it comes to gender differences in AI attitude dimensions, the results are not always significant. For example, Sindermann et al. developed the Attitude Towards Artificial Intelligence (ATAI) scale and found that males scored higher than females in the ATAI Acceptance scale [20]. However, no significant gender differences were found in the ATAI Fear scale. Additionally, the samples from different countries (Germany, the UK, and China) showed significant differences in both ATAI scales, with the Chinese sample having the highest scores in the Acceptance scale and the lowest scores in the Fear scale compared to the samples from Germany and the UK.

Regarding the reasons for gender differences in AI attitudes, Gibert and Valls pointed out that previous research has generally found that women have more negative views on AI compared to men [40]. They suggest that this may be due to a higher representation of men majoring in information-related fields, which reflects different levels of involvement or interest in AI among different genders. Another reason could be that men are generally more optimistic. It was also mentioned that women have more concerns about the application of AI and tend to focus more on social implementation issues related to AI [41].

Whether there are gender differences in the overall ChatGPT attitude scale and individual dimensions is still to be explored.

## 3. Material and Methods

### 3.1. Participants and Procedure

The participants were 516 college students in Taiwan obtained by convenient sampling (350 women and 166 men, mean age = 20.61 with SD = 1.84). A Google Forms link to our questionnaire was posted on SMs (i.e., Facebook and Instagram) and online forums. The present study has been ethically approved by the Institutional Review Board (IRB) of National Chung Cheng University (Ref: CCUREC-112063001).

### 3.2. Measures

#### 3.2.1. Demographic Information and ChatGPT Usage Survey

The questionnaire surveyed the participants’ demographic information, such as gender, age, and major. The ChatGPT (version 3.5) usage survey included the following items:

(1) Weekly usage time and frequency of ChatGPT.
(2) How much are you willing to pay for the advanced version of ChatGPT?
(3) Rate the accuracy of ChatGPT in answering questions on a scale of 1 to 10.
(4) Rate the depth and breadth of ChatGPT’s responses on a scale of 1 to 10.
(5) Overall satisfaction with the interaction with ChatGPT.

#### 3.2.2. The ChatGPT Attitude Scale (CAS)

We developed the CAS based on the structure mentioned Section 2.3. The CAS is a 21-item, 4-point scale. The item description and psychometric properties are illustrated in Section 4.1.

## 4. Results

### 4.1. ChatGPT Usage Survey

The average weekly usage of ChatGPT by participants was 2.16 h (SD = 4.45), and participants were willing to pay TWD 516 (SD = 181.49) for an upgrade, which is approximately USD 16.

In terms of usage frequency, 5.6% used ChatGPT daily, 19.4% used it every two or three days, approximately 24.2% used it once a week, 42.2% used it once a month, and 5.6% never used it. It is clear that about half of the participants use ChatGPT at least once a week.

On a scale of 1 to 10, the average accuracy rating for ChatGPT was 6.61 (SD = 1.94), while the depth and breadth ratings were 6.53 (SD = 1.9). The overall satisfaction rating was 7.08 (SD = 2.00).

### 4.2. Validity and Reliability of the CAS

#### Validity of the CAS

Data Analyses were conducted using the SPSS 27.0 and LISREL 8.80 programs. Factor analysis can broadly be divided into exploratory factor analysis (EFA) and confirmatory factor analysis (CFA); both are common techniques used in scale development, but each serving slightly different purposes [42]. EFA can be used to explore the underlying structures among a set of items and is suitable during the early stages of scale development, while CFA is used to confirm a previously stated theoretical model or factor structures. Since EFA can contribute to model specification prior to cross-validation with CFA [43], some scholars (e.g., Gerbing and Hamilton, Knekta et al., and Yu et al.) [43,44,45] recommend running a two-phase factor analysis by dividing the sample into two halves, using EFA to explore the factor structure of the items for one half and then using CFA to cross-validate the factor structure for the other half.

Therefore, we first randomly split the data into two Group 1 (n = 256) and Group 2 (n = 256). In phase 1, we applied EFA to explore the factor structure and item analysis on Group 1. In phase 2, for (n = 256), we applied CFA on Group 2 to validate the structures obtained from phase 1.

We applied EFA using a Varimax rotation of the principal axis extraction. A value of 0.30 was determined as a viable cut-off point for judging factor loadings. The CAS exhibited a five-T factor structure, named “Tool”,” Threat”, “Tutor”, “Talk”, and “Trend”. The factor loadings of the items are shown in Table 1. This study proposed five factors based on a literature review; the proposed factor structure of this study is supported, as the items and assignment of factors in the EFA’s five-factor solution are reasonable.

**Figure 1:**
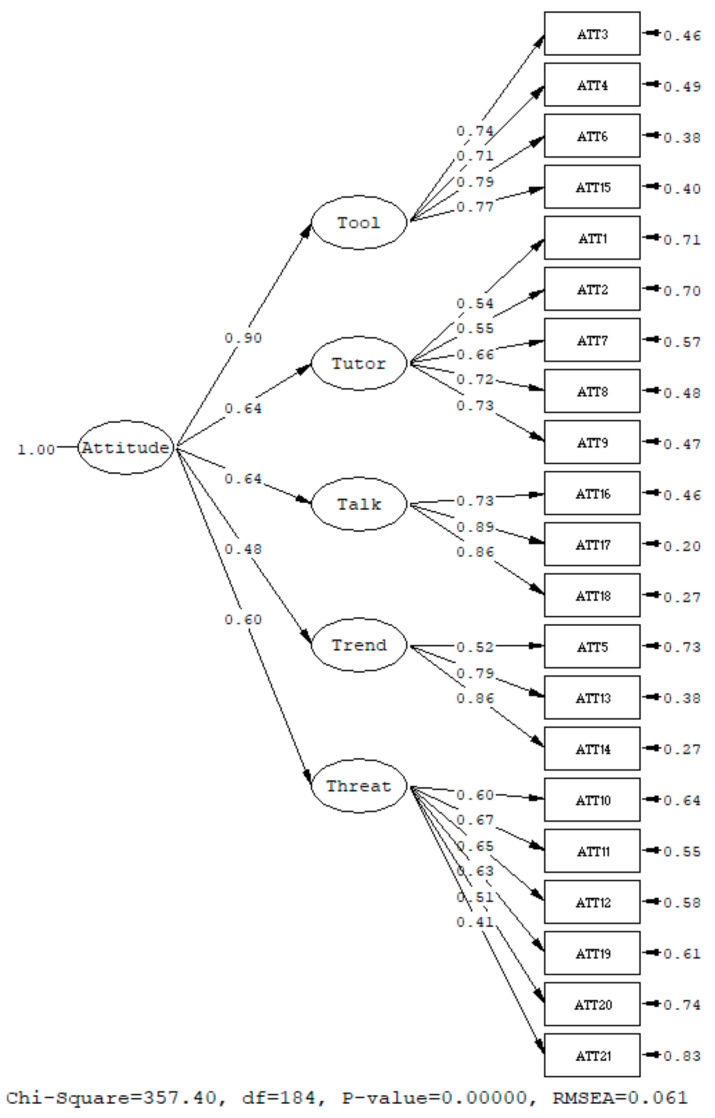
CFA on the CAS with a standardized solution.

Next, a CFA was then run to verify the structure of the CAS obtained from phase 1. Concerning the fit indices, the CFA model showed a good fit (Chi-Square = 357.40, df = 184, *p*-value = 0.00, root mean square error of approximation (RMSEA) = 0.061 < 0.08, 90% CI for RMSEA = (0.051; 0.070), comparative fit index (CFI) = 0.97 > 0.90, standardized root mean square residual (SRMR) = 0.081 < 0.10) [43]. Moreover, for the results of the parameter estimates, all estimates were significant and consistent with the underlying theory. To sum up, the CFA results cross-validated that the CAS exhibited good factor/construct validity. The validity of the five-T (Tool, Threat, Tutor, Talk, and Trend) model of ChatGPT attitude was supported by a CFA. The CFA of CAS showing the standardized solution is shown in Figure 1. For reliability, the Cronbach alpha of the CAS is 0.870, indicating good reliability.

### 4.3. Gender Differences in ChatGPT Attitudes

The results of this study showed that males scored significantly higher than females (*p* < 0.05) on the ChatGPT attitude total scale and the Tool, Tutor, and Trend subscales, as shown in Table 2.

## 5. Discussion

This study developed the CAS based on the 5T (Tool, Tutor, Talk, Trend, and Threat) model. Via an exploratory factor analysis, the 5-T model of CAS was extracted. Moreover, the model was validated via a confirmatory factor analysis. The CAS exhibited good reliability and validity and can be used to explain ChatGPT attitudes.

According to our research findings, university students consider ChatGPT an important “Tool” in their daily lives and a “Tutor” in academics; ChatGPT also serves as a “Talk” feature, offering emotional support. Students recognize ChatGPT as a significant “Trend” of the present era, but they also express considerable concern regarding the possible “Threat” of content falsification and job displacement that it may cause.

Regarding gender differences, males outperformed females in the overall score as well as in the Tool, Tutor, and Trend subscales. This study found that males scored significantly higher than females on the ChatGPT attitude scale. These findings are consistent with previous research on AI attitudes, such as Jang et al., Sindermann et al., and Grassini [14,15,20], which generally found that males have more positive attitudes towards AI. Sindermann et al. measured AI attitudes in samples from Germany, the UK, and China and found that males had more positive attitudes towards AI than females in all three countries [20]. Grassini also found that males hold more positive attitudes towards AI compared to females [12]. Additionally, Pinto dos Santos et al. investigated the attitudes of medical students towards AI in the medical field and found that males had more positive perceptions of the benefits of AI and lower levels of fear towards AI [38].

Regarding the Tool and Tutor dimensions, this study found that for males, ChatGPT is perceived more as a tool and a useful tool for learning new knowledge. In terms of the Trend dimension, this study found that males scored higher than females, indicating that males are more concerned about the technical aspects and view this technology as a future trend. These findings are similar to the results of Horowitz and Kahn [41], who found that males have more positive attitudes towards the utilitarian use of AI, higher acceptance, and more positive attitudes towards future developments.

There were no significant gender differences in the Talk and Threat dimensions. In the Talk dimension, there were no significant differences, indicating that university students of different genders perceive chatbots as having the ability to chat and express emotions. This finding differs from previous research on social media (e.g., Yu et al.) [42], which generally found that females use social media for chatting more. However, in the context of chatbots, there were no significant gender differences. This may be because social media involves social interactions and chats with real people, which involves social skills and interaction anxiety, leading to less utilization by males. But when it comes to chatting with chatbots, there were no significant gender differences in attitudes.

In the Threat dimension, this study found no gender differences in the fear of ChatGPT. This differs from previous research on AI attitudes, where some studies found that males have lower fear towards AI [38,41]. The above studies suggest that males are more optimistic about the development of AI, while females have higher concerns. Males are more focused on the technological aspect of AI development, while females are more inclined to focus on social issues related to AI, leading to anxiety and perceived threats. However, this study found no gender differences in the Threat factor, indicating that both genders perceive potential threats to society from ChatGPT and related AI applications. The powerful and rapid development of AI and the associated threats and social issues have attracted widespread attention regardless of gender. Thus, gender differences in concerns about AI-related threats appear to be less significant among university students compared to previous studies.

## 6. Conclusions

To understand attitudes toward ChatGPT, this study developed the ChatGPT attitude scale (CAS) and recruited a sample of 516 university students from Taiwan for the research. The results of the exploratory factor analysis revealed that the CAS exhibited a five-factor structure, named “Tool”, “Threat”, “Tutor”, “Talk”, and “Trend”. The confirmatory factor analysis validated the 5-T (Tool, Tutor, Talk, Trend, and Threat) model, which can be used to explain the corresponding five factors that constitute ChatGPT attitudes.

Regarding gender differences, compared to females, males scored higher on the total scale and the Tool, Tutor, and Trend subscales, while there were no significant differences in the Talk and Threat subscales.

This study has several limitations. Firstly, in terms of sample, this study sampled university students from Taiwan. Although the scale demonstrated good reliability and validity, further cross-cultural validation is needed to obtain more cross-cultural confirmation. Additionally, in terms of sample gender composition, this study had a higher proportion of females compared to males. This phenomenon is common in many survey studies (e.g., Yu, Sindermann et al.) [20,42], and it may be because females are more willing to respond to questionnaires [42].

Furthermore, the development of chatbots is rapid, and this study was conducted only in the second quarter of 2023. Considering the ongoing changes in technological development and usage behavior, future research should conduct longitudinal studies to understand the changes in attitudes and usage behavior towards chatbots.

Additionally, in terms of software categories, the application of chatbots may extend beyond the ChatGPT 3.5 software. Other AI tools (e.g., Midjourney, ChatSonic, and HuggingFace) are also worth researching as their user base grows.

## Figures and Tables

**Table 1:** Factor loadings for EFA of the CAS.

	Factor Loading				
	1	2	3	4	5
**Factor 1: Tool**					
I use ChatGPT to search for current news or the latest trends.	0.742	0.133	0.187	0.186	0.147
I use ChatGPT to plan travel itineraries or recommend food.	0.734	0.177	0.159	0.188	0.072
I use ChatGPT to understand current trends.	0.729	0.241	0.154	0.218	0.104
I use ChatGPT to provide information on various topics, such as the news and weather.	0.705	0.176	0.111	0.202	0.075
**Factor 2: Tutor**					
I use ChatGPT to learn skills like writing, programming, sales, etc.	0.069	0.781	0.032	0.160	0.177
I use ChatGPT to learn subject knowledge, such as history, mathematics, computer science, etc.	0.146	0.730	0.086	0.170	0.173
I use ChatGPT to learn foreign languages.	0.367	0.675	0.104	0.129	−0.028
I use ChatGPT to handle work or academic problems.	0.006	0.636	−0.009	−0.030	0.460
I use ChatGPT for language translation.	0.290	0.620	0.067	−0.060	0.145
**Factor 3: Threat**					
With the rapid advancement of AI technologies like ChatGPT, everyone should exercise caution.	0.046	−0.010	0.735	0.029	0.227
I’m concerned that ChatGPT might leak my conversation records.	0.248	0.031	0.673	0.129	−0.035
I’m afraid that ChatGPT will replace my job.	0.039	0.189	0.656	0.104	0.039
Due to ChatGPT, the problem of plagiarism in student assignments or papers may become more serious.	0.153	0.021	0.597	−0.119	0.252
ChatGPT is unreliable.	−0.001	0.011	0.596	0.088	−0.187
I worry that ChatGPT will decrease my interpersonal interactions.	0.374	0.038	0.596	0.255	−0.116
**Factor 4: Talk**					
I ask ChatGPT about emotional or interpersonal issues.	0.199	0.114	0.142	0.866	0.050
I discuss personal growth-related questions with ChatGPT.	0.209	0.133	0.116	0.838	0.115
I chat with ChatGPT to pass the time or alleviate loneliness.	0.343	0.061	0.085	0.749	0.022
**Factor 5: Trend**					
I believe using ChatGPT is the future.	0.030	0.170	0.087	0.015	0.838
I think ChatGPT is a powerful force driving change in various industries.	0.113	0.242	0.117	0.066	0.807
I find ChatGPT to be interesting and enjoyable.	0.323	0.224	−0.150	0.209	0.565

**Table 2:** Summary of gender differences in the CAS scores.

Measure	Men		Women		t-Value
	M	SD	M	SD	
Attitude	50.78	10.16	48.04	11.14	2.69 **
Tool	8.59	3.16	7.85	3.32	2.42 *
Threat	13.19	3.86	13.19	3.77	0.00
Tutor	13.60	3.58	12.26	4.01	3.66 ***
Talk	5.49	2.49	5.41	2.75	0.33
Trend	9.92	1.64	9.34	2.07	3.44 **

Note. * *p* < 0.05. ** *p* < 0.01. *** *p* < 0.001.

## Data Availability

The datasets generated by the survey research during and/or analyzed during the current study are available in the Dataverse repository, https://www.dropbox.com/scl/fi/sbipeuptzk0g810fwfx0b/attitude-opendata-ascii.txt.prn?rlkey=qzbn2h9em4klpk8auikvfmx6v&dl=0 (accessed on 12 July 2024).
